# Team-based learning (TBL) in clinical disciplines for undergraduate medical students—a scoping review

**DOI:** 10.1186/s12909-023-04975-x

**Published:** 2024-01-03

**Authors:** Irene Sterpu, Lotta Herling, Jonas Nordquist, Jerome Rotgans, Ganesh Acharya

**Affiliations:** 1 Division of Obstetrics and Gynecology, Department of Clinical Sciences, Intervention and Technology (CLINTEC), Karolinska Institutet, Stockholm, Sweden; 2 Center for Fetal Medicine, Pregnancy Care and Delivery, Karolinska University Hospital, Stockholm, Sweden; 3 Department of Medicine (Huddinge), Karolinska Institutet, Stockholm, Sweden; 4 Department of Clinical Medicine, UiT The Arctic University of Norway, Tromsø, Norway

**Keywords:** Team-based learning, Clinical disciplines, Undergraduate medical education, Active learning

## Abstract

**Background:**

Team-based learning (TBL) is an evidence-based pedagogical method that has been used in undergraduate medical education since 2001. However, its use in clinical disciplines is rarely reported, and the impact of its implementation is not known. The aim of this study was to explore and map the published literature on the impact of implementing TBL in clinical disciplines in undergraduate medical education.

**Methods:**

A comprehensive search of Medline, Education Resources Information Center (ERIC), and Web of Science databases was performed on November 24, 2021 and updated April 6, 2023, using relevant Medical Subject Headings (MeSH) and free-text terms. Original research studies reporting on the implementation of TBL in clinical disciplines in undergraduate medical education published in peer-reviewed English language journals were included irrespective of their methodological design.

**Results:**

The initial search identified 2,383 records. Of these, 49 met the inclusion criteria. Most of the studies (*n* = 44, 90%) described the implementation of a modified version of TBL in which one or more TBL steps were missing, and one study had undefined protocol for the implementation. The most reported outcomes were knowledge acquisition (*n* = 38, 78%) and students’ satisfaction or attitudes toward TBL (*n* = 34, 69%). Despite some differences in their results, the studies found that implementing TBL is associated with increased knowledge acquisition (*n* = 19, 39%), student engagement (*n* = 6, 12%), and student satisfaction (*n* = 31, 63%).

**Conclusions:**

Most of the studies reported positive results in students’ satisfaction and students’ engagement, whilst the results on knowledge acquisition and retention were more contradictory. In most of the studies, TBL was implemented in a modified form and diverse comparators were used. The methodological quality also varied. Thus, no unequivocal conclusions could be drawn regarding the value of implementing TBL in clinical disciplines. More studies with rigorous methodologies are needed in this field.

**Supplementary Information:**

The online version contains supplementary material available at 10.1186/s12909-023-04975-x.

## Background

Team-based learning (TBL) was introduced by Larry Michaelsen in the 1970s in business education and was adapted to medical education in the early 2000s [1]. Following its introduction, TBL quickly gained popularity in medical schools across the United States and then internationally [2, 3]. The global adoption of TBL has been widespread, with medical schools across diverse healthcare systems, including the United States, Canada, the United Kingdom, Australia, and several Asian countries, such as Singapore. The adaptation of TBL addressed the challenges of accommodating increasing class sizes and the need for more engaging learning methods in medical education. In addition, TBL was recognized for fostering critical thinking, application of knowledge, and teamwork [4, 5]. The body of literature, including comparative studies with traditional and other active-learning methods, provides growing evidence of TBL's effectiveness in improving knowledge retention, student satisfaction, and academic performance [6–9].

An advantage of TBL over other active-learning strategies, such as problem-based learning (PBL), is its unique structure and combination of small and large class interactions. It allows for small group discussions (5–6 students), while being conducted within a large class setting that allows the entire class to engage collectively in the TBL session. This integration of individual preparation and team collaboration into a single class session makes TBL both resource-efficient and manageable—in terms of planning and scheduling—but also addresses some of the limitations commonly associated with PBL [10]. For instance, PBL often requires significant faculty time for facilitation and can be challenging to scale for larger classes. In contrast, TBL, facilitates a more scalable active-learning environment that can accommodate larger student numbers without proportionally increasing the demand on faculty time [11, 12]. Furthermore, the structured readiness assurance process and the immediate feedback mechanism inherent to TBL provide a more standardized assessment of student preparedness and engagement than PBL. These elements of TBL contribute to its possible learning effectiveness and at the same time enhancing administrative efficiency in medical education settings [12, 13].

While the benefits of TBL in undergraduate health-care education are well-documented with numerous reports of higher examination scores, student engagement, and student satisfaction [1, 14–17], its application has predominantly been restricted to the preclinical settings such as embryology, anatomy [18, 19]. Relatively few studies have been conducted within the clinical years of medical education, where the nature of learning shifts significantly from theoretical, conceptual knowledge towards practical, patient-centered skills and decision-making in real-world medical scenarios. The effectiveness of TBL in this context is less explored, with limited evidence on whether the benefits observed in preclinical settings translate to the clinical environment. This gap is important, as the demands of clinical education differ markedly from preclinical education, and teaching strategies that are effective in one may not have an impact in the other.

The aim of this study was to explore and map the published literature on the impact of implementing TBL in clinical disciplines in undergraduate medical education for the purpose of synthesizing existing evidence and identifying research gaps.

## Methods

A scoping review was conducted according to the guidance document of the Joanna Briggs Institute [20] and earlier work by Arksey and O’Malley [21]. The results have been analyzed and reported following the Preferred Reporting Items for Systematic reviews and Meta-Analyses (PRISMA) extension for Scoping Reviews guidelines [22].

### Stage 1: the research question

The primary research question was: “Where and how is TBL implemented in clinical disciplines in undergraduate medical education?” and the secondary question “What outcomes are measured and how are they measured?

### Stage 2: search strategy and identifying relevant studies

An electronic literature search of the following databases was performed: Medline, ERIC, and Web of Science. After the original search was conducted on November 24, 2021, the search was updated April 6, 2023. The search strategy was developed in Medline (OVID) in collaboration with librarians at the Karolinska Institutet University Library. For each search concept, Medical Subject Headings (MeSH terms) and free-text terms were identified. The search terms included “team-based learning,” “tbl + learning”. The same search terms were then used in the other databases. The strategies were peer reviewed by a second librarian prior to execution. No language restriction was applied, and the databases were searched from inception. Subsequently, duplicates were removed using the method described by Bramer et al. [23]. The full search strategies for all the databases are available in Additional file 1.

### Stage 3: study selection

Studies were eligible for inclusion if they were peer-reviewed educational studies that evaluated the impact of implementing TBL in clinical disciplines in undergraduate medical education, irrespective of their methodological design (i.e. quantitative, qualitative, and mixed methods studies were eligible). Studies were considered for inclusion regardless of whether full-concept TBL or modified TBL (using only some components) was examined. The exclusion criteria were: TBL was implemented in preclinical disciplines for undergraduate medical students; TBL was implemented in postgraduate education; the study was published in a language other than English; or the study was a review article, editorial, commentary, guideline, conference abstract or expert opinion.

### Stage 4: data extraction

After the search, the first author screened the titles and abstracts against the inclusion criteria for the scoping review. Two authors (IS and LH) then assessed the full text of each potentially relevant article for eligibility. Eight articles were assessed by a third author (GA) since it was unclear if the discipline was preclinical or clinical, the implementation of TBL was not described or the intervention was not real TBL. The disagreement was resolved by reassessing the article, discussing it and a consensus was reached. We tried to be more inclusive when assessing the studies. In adherence with the inclusion criteria, two authors (IS and LH) independently extracted the data and recorded it on an Excel spreadsheet. Descriptive statistics were calculated to summarize the data. Frequencies and percentages were utilized to describe nominal data. The extracted data included the author(s), publication year, study design, country, population, name of the clinical discipline, teaching method in the control group (if applicable), outcomes reported, method(s) used to assess the outcomes and TBL components implemented. The PICO framework was used for reporting the results of the scoping review in Supplementary Table 1: Population (the medical students, year, and number), Intervention (the type of TBL implemented, modified version or TBL), Comparison (if there were any comparators and which type of pedagogical comparators were used) and Outcomes.

Typically, TBL unfolds across three distinct phases [24–27]. The initial phase is dedicated to individual study, where students independently review materials such as video lectures, textbook chapters, scholarly articles, or digital content assigned by their instructor [28]. This self-directed learning phase is critical for setting the groundwork for in-class activities. Once in class, the process transitions to the second phase, the readiness assurance phase [29]. This begins with students individually completing a closed-book quiz (known as the individual readiness assurance test, or iRAT) to assess their grasp of the study materials. The iRAT usually contains 15 to 25 multiple-choice questions. Following the iRAT, students convene in small groups of 5–7 (the team readiness assurance test, or tRAT) to retake the same test in collaboration. During the tRAT, group dialogue is encouraged as students debate over each question, consolidating their collective answer before submission. After the responses are submitted, the correct answers are revealed, often initiating further inquiry into the topic, sometimes referred to as "appeals" or "burning questions", at which point the instructor steps in to provide further explanations [2]. The final phase is the application exercise [24]. In this phase, the small groups tackle a series of real-life exercises encouraging them to apply what they have learned to a concrete medical context. These application exercises are pivotal, as they compel students to implement their learning in realistic and contextually relevant situations [10, 30]. See Table 1 for an overview of the steps involved.

**Table 1:** Overview of the TBL steps

Step	Description
1. Pre-Assignment	Independent completion of preparatory work before class session
2. iRAT	An individual quiz to assess understanding and learning of the pre-class material
3. tRAT	The same quiz taken by teams to facilitate discussion and ensure team preparedness
4. Appication Exercise	Collaborative problem-solving exercises that apply course concepts
5. Peer Review	Feed-back provided by students on their peers’ contribution to team activities

## Results

### Selection of studies

**Fig. 1:**
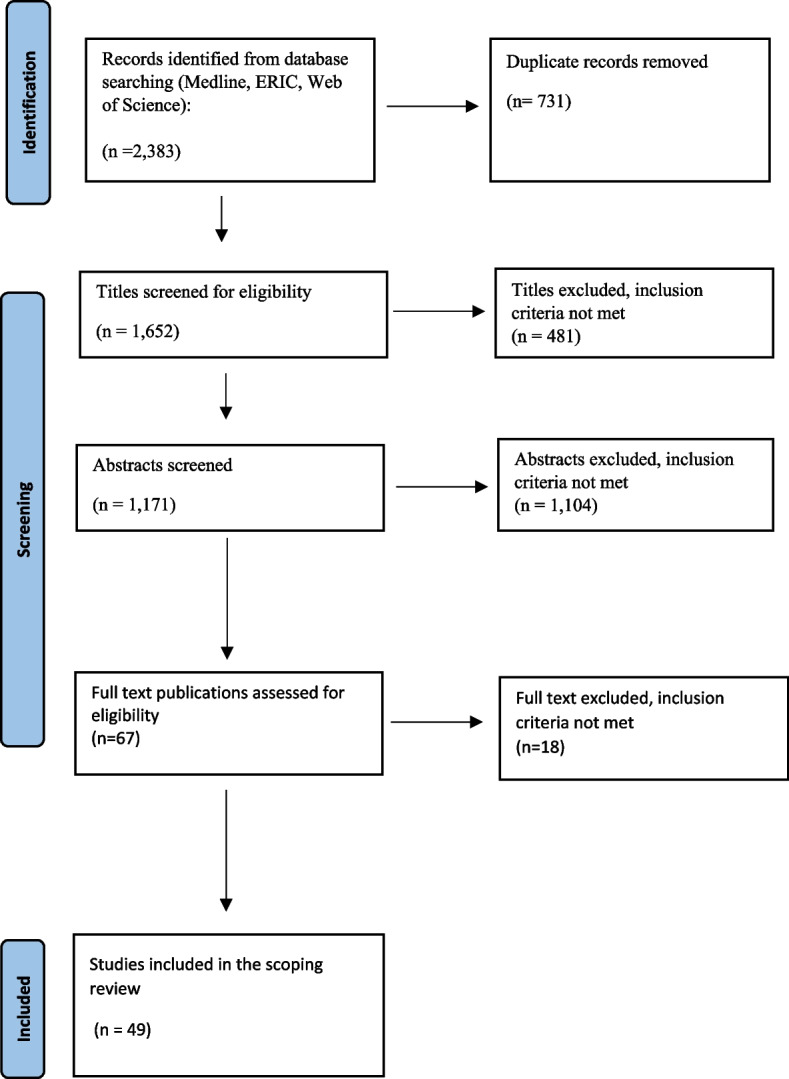
Study flow PRISMA diagram. Includes the number of records identified, included and excluded

The process used to select the studies is presented as a PRISMA flow chart in Fig. 1 and in Additional file 1. After conducting the systematic literature search and removing duplicate articles, 1,652 articles were identified as potentially relevant. Of these, 1,585 were excluded after screening the titles and abstracts. A total of 67 full-text articles were assessed for eligibility, and 18 were excluded because after being assessed by both IS and LH. The detailed reasons for exclusion are provided in Fig. 1 and Additional file 1. This left 49 studies that met the prespecified inclusion criteria. A manual search of the reference lists of the 49 articles did not yield additional articles that met the inclusion criteria.

### Characteristics of the included studies

The publication dates of the articles included in the review (*n* = 49) ranged from 2004 to 2022. The main characteristics of these studies are summarized in Table 2 and the detailed results are also available in Supplementary Table 1 in the Additional file 2. The studies were conducted in different countries, with the vast majority conducted in North America (22/49), followed by Asia (18/49) and Europe (8/49).

**Table 2:** Characteristics of the 49 publications included in the scoping review

Characteristics of the publications	Number(percentage)
**Year of publication**	
2001–2011	4 (8%)
2012–2022	45 (92%)
**Continents and countries**	
**Africa**	
⦁ Sudan (1)	1 (2%)
**Asia**	
⦁ China (6), Saudi Arabia (3), Iran (2), Singapore (2), Egypt (1), Lebanon (1), United Arab Emirates (1), Pakistan (1,) Oman (1)	18 (37%)
**Europe**	
⦁ Germany (4), Turkey (2), UK (1), Finland (1)	8 (16%)
**North America**	
⦁ United States of America (20) and Canada (2)	22 (45%)
**Clinical disciplines:**	
⦁ Neurology	8 (16%)
⦁ Ophthalmology	5 (10%)
⦁ Pediatrics	5 (10%)
⦁ Psychiatry	4 (8%)
⦁ Obstetrics and gynecology	3 (6%)
⦁ Emergency medicine	3 (6%)
⦁ Intern medicine	3 (6%)
⦁ Other^a^	18 (37%)
**TBL implementation**	
⦁ Modified TBL	44 (90%)
⦁ Whole TBL	4 (8%)
⦁ Not described	1 (2%)
**Comparator**	
⦁ Without Comparator	18 (37%)
⦁ Lecture-based learning	19 (39%)
⦁ Seminars	4 (8%)
⦁ Other^b^	8 (16%)
**Outcomes reported**	
⦁ Knowledge acquisition and retention	38 (78%)
⦁ Students ‘satisfaction/experience	34 (69%)
⦁ Students’ engagement	6 (12%)
⦁ Teamwork and team interaction	5 (10%)
⦁ Clinical decision-making skills	4 (8%)
⦁ Students’ learning style	1 (2%)
⦁ Script concordance test	1 (2%)
⦁ Teacher’s attitude	1 (2%)
	1 (2%)

^a^Family medicine (2), Surgery (2), Hematology (2), Rheumatology (1), Endocrinology (1), Ambulatory medicine (1), Dermatology (1), Occupational medicine (1), Community medicine (1), Immunology (1), Ultrasound skills (1), Clinical and communications skills (1), Prescribing safety assessment (1), Clinical medicine (1), Topics from different clinical disciplines (1)

^b^Combined pedagogical methods: CBD and lecture (2), Peer-assisted learning vs conventional teaching (1), self-studies, passive learning (2), pre-implementation condition (1), teaching rounds (1), online TBL (1)

In the included studies, the number of students participating in the TBL sessions ranged from 11 to 484 medical undergraduate students and varied from small groups to entire year groups. The clinical disciplines in which TBL was most implemented were neurology (8 studies), ophthalmology (5 studies), psychiatry (4 studies) obstetrics and gynecology (3 studies), pediatrics (3 studies) and emergency medicine (3 studies). The remainder of the studies implemented TBL in a range of other disciplines.

In 32 studies, the implementation of TBL was compared to 1) other educational methods, such as the delivery of lectures (20 studies) [31–50] and seminars (4 studies) [51–54]; 2) combined pedagogical methods, such as case-based discussion and lecture delivery, [55, 56] peer-assisted learning and conventional teaching [57], self-reading and passive learning [58, 59]; 3) the pre-implementation condition [60] 4) teaching rounds [4] and 4) online TBL [61]. Seventeen (35%) of the included studies had no comparator [50, 62–78].

Forty-four of the 49 studies implemented modified TBL, with one or more of the four steps originally described by Michaelsen [30] missing or not described in the methodology. In one study the TBL steps were not described. There were only four studies that described implementation of TBL as a full concept in clinical disciplines The peer-review step performed at the end of the TBL session was missing or not described in 39 studies.

### Outcomes of the included studies

The measured outcomes of the implemented TBL methodologies varied (summarized in Table 2, along with the assessment instruments/tools. The most common outcomes reported were academic performance and knowledge acquisition/retention (38 studies), students’ satisfaction/experience with TBL (34 studies), students’ engagement (6 studies), teamwork and team interaction (5 studies), clinical decision-making skills (4 studies), students’ learning style (1 study), script concordance test (1 study) and teachers’ attitude toward TBL (1 study).

### Knowledge acquisition/retention

The methods used to assess knowledge acquisition and retention varied from knowledge tests after the TBL sessions to final exam grades or even national board exam scores. Twenty-three studies reported that students who participated in TBL had either higher final exam grades, mean scores on knowledge tests, or board exam scores. Mayel et al. [41] showed that there was no difference between the pretest scores of groups who did and did not participate in TBL and that there were higher posttest scores among the group who participated in TBL. Krase et al. [31] reported improved national board mean score results among TBL participants; however, there was no difference in their knowledge retention compared to a non-TBL group. Similarly, Langer et al. [53] found that there was improved knowledge after a TBL course but no difference in long-term knowledge retention compared to the non-TBL group.

Two studies [72, 73] reported higher scores in tRAT compared to iRAT as their only knowledge results.

In contrast, Mody et al. [35], Langer et al. [69], Birch et al. [51], Jost et al. [52] and Alimoglu et al. [32] found no differences in scores in knowledge tests/final exams in the TBL group. However, the latter two reported that TBL groups performed better in key-feature problem examination and had higher knowledge retention. In addition, Kaminski et al. [33] reported lower national board scores after the implementation of TBL, and Larchenfeldt et al. [70] found that tRAT averages were comparable over a period of three years in the TBL groups.

### Students’ satisfaction and attitudes toward TBL

Among the 49 articles, 34 reported on outcomes, such as students’ satisfaction with TBL or collected data on students’ experiences with and attitudes toward TBL. More than half of these 34 studies (*n* = 21) compared TBL to traditional teaching methods, while the rest (*n* = 13) had no comparator. Thirty-one of the studies reported that students had positive attitudes toward TBL and a high level of satisfaction with the TBL sessions or that TBL facilitated deeper learning, was better at fulfilling learning objectives, or was a valuable experience according to students’ self-reporting.

Two studies, conducted by Krase et al. and Thomas et al. [31, 37], found that there were no differences in students’ satisfaction between TBL vs non-TBL groups. Furthermore, in the study of Omer et al. [71], students indicated a low preference for TBL, poor satisfaction with TBL, and low TBL ratings. This might be partially explained by the traditional teaching method being a combination of methods, such as didactic lectures, bedside teaching, and simulations.

### Students’ engagement

Alimoglu et al. [32] assessed student engagement via observation and in-class engagement measures based on an observational tool called STROBE [79]. The mean in-class engagement scores were significantly higher for TBL groups, both for learners and instructors.

Three studies reported a higher level of engagement when TBL was utilized compared to traditional teaching methods [34, 38, 56], and two studies reported high student engagement in TBL sessions; however, they did not include a comparator [58, 62].

### Teamwork and team interaction

Team emotional intelligence was assessed using the Workgroup Emotional Intelligence Profile (WEIP-S) in two of the 49 studies. Both studies found that team emotional intelligence was higher post-clerkship compared to pre-clerkship [60, 63]. When control groups were included, it was found that TBL resulted in significantly higher gains in areas such as awareness of one’s own emotions, recognizing emotions in others, and ability to manage others’ emotions [60].

Levine et al. [56] studied the value of learning in teams by using the Classroom Engagement Survey developed at Baylor College of Medicine and the Value of Teams instrument, a 17-point survey used to evaluate working in groups and working with peers. Their results showed that those who participated in TBL sessions showed significant improvement in the “value of working with peers” and “value of group work” compared to historical cohorts from previous years. Warrier et al. [38] found that there was no significant difference in the overall “value of working with peers” pre and post-TBL. However, they found that there was a significant difference in the “value of group work” in those who had participated in TBL.

Finally, Field et al. [66] examined students’ attitudes toward teamwork. They found significant improvements in the areas of “satisfaction with team experience,” “team impact quality of learning,” and “team impact on clinical reasoning ability” after the team-based revision (TBR) sessions. The TBR did not involve a preparation phase and was based on students’ acquired knowledge during previous clinical rotations.

### Clinical decision-making skills

In a study conducted by Jost et al. [52], an intervention group was subjected to a supplementary TBL class on clinical decision-making skills and a control group was not. The participants’ clinical decision-making skills were assessed via a key-feature problem examination where the intervention group performed significantly better than the control group.

Ong et al. [36] studied the neurological clinical reasoning associated with TBL using a validated Script Concordance Test (SCT). In a neuroanatomical localization seminar, the SCT scores of TBL participants were significantly higher than those who participated in interactive lectures. In neurological emergencies seminar, the SCT scores of TBL participants were similar to those of participants who attended interactive lectures.

Abouzeid et al. [77] found a significant difference between the students’ and experts’ whole test scores and their scores on most of the vignettes. However, when the test was completed in teams, the scores for 9 out of the 17 vignettes showed non-significant differences with the experts’ scores on these vignettes.

### Students’ learning styles

Cremerius et al. [57] assessed the effect of TBL on students’ learning styles and utilized Kolb’s Learning Style Inventory for this purpose. They found that the learning style had a significant impact on the students’ practical performance in all groups.

### Teachers’ attitudes toward TBL

One of the studies included in this review investigated teaching effort and teachers’ attitudes toward TBL [40]. In this study, the teachers (the number of teachers interviewed was not specified) reported that TBL was associated with having a higher class-preparation workload than lecture-based teaching (12 h vs. 5 h). However, this was expected since TBL was a recently introduced methodology. The teachers also reported that the atmosphere in the classroom was more engaging and active when TBL was used.

## Discussion

### Main findings

To our knowledge this is the first scoping review focusing on this topic. This review demonstrated that only four out of 49 (8%) articles described the implementation of the complete TBL concept in clinical disciplines in medical education. The most reported outcomes in this review were students’ satisfaction and attitudes toward TBL (*n* = 34) and/or students’ knowledge acquisition (*n* = 38). Most studies (*n* = 31) reported positive findings, with a high level of student satisfaction. The studies that had student engagement as an outcome (*n* = 6) reported all a high level of students’ engagement with TBL.

### Interpretation of results

Relatively few TBL-based studies have been conducted in clinical disciplines compared to preclinical disciplines, and most were published between 2017 and 2022 (33 studies). Because only a low number of publications have focused solely on clinical disciplines, and due to our wish to conduct an inclusive review, we included studies that covered clinical disciplines in preclinical courses. Neurology was found to be the leading discipline in which TBL is often used, and this could be partially explained by the fact that students rate neurology as one of the most difficult disciplines. [80, 81] Fewer studies have evaluated the use of TBL in surgical specialties, and this could be because surgical skills are mostly taught in simulation settings, and in operating rooms [30, 44].

Most of the studies included in this review were conducted in North America (*n* = 22), and a limited number described the European experience of implementing TBL in clinical disciplines (*n* = 8). It is possible that this line of research is in its infancy and just gaining momentum. It is also worth noting that in preclinical education, there is uniformity in the type of teaching, with lectures being the main teaching method, whereas in clinical education, the teaching methods tend to be more complex, using a combination of lectures, seminars, case-based discussions, and clinical rotations with bedside education. This difference could partially explain the multiple comparators used in the studies included in this review.

There is a variation in the outcomes reported in the examined studies; however, most of the studies focused on student knowledge, perception, and satisfaction. Student learning was assessed in various ways, from analyzing iRAT and tRAT results to final exam scores, pretest and posttest results, or national board exams scores, which made it difficult to compare the results of different studies.

While most of the studies showed that knowledge increased after undertaking a TBL module, Langer et al. [53] and Krase et al. [31] found that there was no difference in long-term knowledge retention. This finding is quite surprising since TBL is considered an active-learning method with application exercises designed to promote a deeper understanding of the subject.

The results of a meta-analysis of the effect of TBL on content knowledge showed that there was a moderate positive effect in studies of both undergraduate- and graduate-level education in pharmacy and medicine [82]. The impact of TBL sessions on knowledge acquisition can vary depending on the context, learning environment, number of sessions per course, ability of the facilitator, and quality of the application exercises. Therefore, we want to highlight the importance of training faculty in the design and delivery of TBL sessions, including the creation of meaningful application exercises that support learning [11, 83].

Students’ satisfaction with and attitudes toward TBL were reported as outcomes in 34 studies. Most (*n* = 31) reported positive findings, with a high level of student satisfaction. However, since more than half of these studies had no comparator, and three studies showed no difference in satisfaction or less satisfaction with TBL, it is difficult to draw firm conclusions about students’ satisfaction with TBL. To understand these contrasting results, we must consider the bigger picture of clinical disciplines. Clinical rotation is usually very short and do not allow students sufficient time to become familiar with TBL [84]. The differences in the results could also be partially explained by how familiar the TBL instructors are with the teaching method, as discussed by Sharna et al. [85]. Also, the instructors’ experiences were not always reported in the studies included in this review. Implementing TBL requires a big shift in the learners’ and teachers’ roles, with the learners having a more active role and the teachers becoming facilitators [86]. These changes, together with the learners’ expectations, can also contribute to the divergent results. A study conducted in a nursing education context showed that there can be improvement over time; the authors reported that there was increased student acceptance of TBL and improved perceptions, suggesting that an adjustment period may be necessary [87]. In this review, only six studies were found that reported student engagement as an outcome, and all reported higher student engagement when TBL was used, which is not surprising, considering the methodology of TBL.

Due to differences in the rigorousness of the studies’ methodology, the extent to which the TBL interventions were described, the versions of TBL implemented (with one or more steps missing), and the comparators used, it was difficult to evaluate the benefits of TBL in clinical disciplines. Therefore, there is clearly a need for methodologically rigorous and well-planned studies in clinical disciplines in which the TBL concept is applied as a whole to gain a deeper understanding of the value of TBL in clinical disciplines.

## Strengths and limitations

To our knowledge, this is the first scoping review focusing on the implementation and outcomes of TBL in clinical disciplines in undergraduate medical education. In addition, adherence to the core TBL elements was scrutinized, and we conducted and reported this review according to existing contemporary methodological frameworks [21, 22]. We decided to adopt a comprehensive search strategy using broad search terms in three electronic bibliographic databases. An additional search was conducted on April 6 for updating the timelines of this review. Our efforts were to be as comprehensive as possible. The quality of the studies included is varying with only a few studies with high evidence methodology. The type of education and training in TBL methodology for the TBL facilitators was not always described in the studies and this factor could affect the outcome of TBL.

The scoping review was limited to peer-reviewed articles published in the English language, and preprint servers, theses repositories, and gray literature were not searched. Therefore, there might be other relevant studies on this subject that were not captured during our literature search.

Another limitation is the risk of publication bias, where articles that report no differences between teaching methods or negative results are not published which could have influenced the results.

## Conclusions

In this scoping review we explored and mapped the implementation of TBL in clinical disciplines in undergraduate medical education. Few studies describe the implementation of TBL as a full concept in clinical disciplines in undergraduate medical education; in fact, only four were identified in this review. Most of the studies reported positive results in students’ satisfaction and students’ engagement. The studies used diverse comparators and varied in methodological quality, making it difficult to really assess the value of TBL in clinical disciplines. More studies with rigorous methodologies and where the implementation of TBL is compared with interactive seminars are needed in this field.

### Supplementary Information

**Additional file 1.**

**Additional file 2.**

## Data Availability

All data are available from the corresponding author on reasonable request.

## Abbreviations

iRAT: Individual readiness assurance test
ERIC: Education Resources Information Center
SCT: Script Concordance Test
TBL: Team-based learning
tRAT: Team readiness assurance test
WEIP: Workgroup Emotional Intelligence Profile
