# Supplementary material for: Team-based learning (TBL) in clinical disciplines for undergraduate medical students—a scoping review
Source: BMC Med Educ. 2024 Jan 3;24:18. doi: 10.1186/s12909-023-04975-x (PMC10765894; doi:10.1186/s12909-023-04975-x)
Supplement: Supplementary file 1 — Additional file 1. [file 12909_2023_4975_MOESM1_ESM.docx]

Documentation of search strategy

University Library search consultation group

Date: November 24, 2021. Search repeated April 6 2023.

Topic/research question: Team-based learning/implementation of team-based learning

Name of researcher(s): Irene Sterpu, Lotta Herling, Jonas Nordqvist, Jerome Rotgens, Ganesh Acharya

Librarian(s): Jonas Pettersson & Anja Vikingson & Lovisa Liljegren

Databases:

1. Medline (Ovid)
2. ERIC (Proquest)
3. Web of Science (Clarivate)

Total number of hits:

- Before deduplication: 2,383
- After deduplication: 1,652

PRISMA 2020 flow diagram for new systematic reviews, including searches of databases and registers only^[[1]](#footnote-1)^

**Identification of studies via databases and registers**

Records removed *before screening*:

Duplicate records removed (n = 731)

Records identified from*:

Databases (n =2,383)

**Identification**

Records screened

(n = 1,652)

Records excluded*

(n = 442)

Reports sought for retrieval

(n = 1,210)

Reports not retrieved**

(n = 39)

**Screening**

Reports assessed for eligibility

(n = 1,171)

Reports excluded:

Reason 1 (n = 295)

Reason 2 (n = 248)

Reason 3 (n = 59)

Reason 4 (n = 93)

Reason 5: (n = 159)

Reason 6: (n = 250)

Studies initially included

(n = 67)

Reports excluded after reading by IS and LH: (n= 18) ***

**Included**

Reports included after discussion and assessment

(n = 49)

* Records were excluded because they were not team-based learning (TBL)-related.

**Records were excluded because they were conference abstracts or in languages other than English.

Reason 1: Other healthcare professions

Reason 2: Preclinical disciplines

Reason 3: Duplicate

Reason 4: Postgraduate education

Reason 5: Methodology article

Reason 6: Non-medical education

*** 8 reports (of 67) were assessed and discussed with a third author (GA) and after discussion 2 were excluded and 6 were included.

1.Page MJ, McKenzie JE, Bossuyt PM, Boutron I, Hoffmann TC, Mulrow CD, et al. The PRISMA 2020 statement: An updated guideline for reporting systematic reviews. BMJ 2021;372:n71. doi: 10.1136/bmj.n71

1. Medline

| Interface: Ovid MEDLINE(R) and Epub Ahead of Print, In-Process & Other Non-Indexed Citations and Daily  Date of Search: April 6, 2023  Number of hits: 869  Comment: In Ovid, two or more words are automatically searched as phrases; i.e. no quotation marks are needed | Field labels   - exp/ = exploded MeSH term - / = non exploded MeSH term - .ti,ab,kf. = title, abstract and author keywords - adjx = within x words, regardless of order - * = truncation of word for alternate endings |
| --- | --- |
| \| **#** \| **Searches** \| **Results** \| \| --- \| --- \| --- \| \| 1 \| team based learning.ab,kf,ti. \| 863 \| \| 2 \| (tbl adj2 learning).ab,kf,ti. \| 441 \| \| 3 \| 1 or 2 \| 869 \| | |

2. Eric

| Interface: ProQuest  Date of Search: April 6, 2023  Number of hits: 339 | Field labels   - ti,ab,kw = title, abstract and author keywords - NEAR/x = within x words, regardless of order - * = truncation of word for alternate endings |
| --- | --- |
| (ti("team based learning") OR ab("team based learning") OR ti(tbl) OR ab(tbl)) 339 | |

3. Web of Science Core Collection

| Interface: Clarivate Analytics  Date of Search: April 6, 2023  Number of hits: 1175 | Field labels   - TS/Topic = title, abstract, author keywords and Keywords Plus - NEAR/x = within x words, regardless of order - * = truncation of word for alternate endings   Note: sometimes “quotation marks” are needed for single search terms to avoid automatic term mapping (lemmatization). |
| --- | --- |
| \| Search No \| Search \| Result \| \| --- \| --- \| --- \| \| 1 \| (TS=("team based learning")) OR TS=(tbl NEAR/2 learning) \| 1175 \| | |

1. [↑](#footnote-ref-1)
