# Supplementary material for: Team-based learning (TBL) in clinical disciplines for undergraduate medical students—a scoping review
Source: BMC Med Educ. 2024 Jan 3;24:18. doi: 10.1186/s12909-023-04975-x (PMC10765894; doi:10.1186/s12909-023-04975-x)
Supplement: Supplementary file 2 — Additional file 2. [file 12909_2023_4975_MOESM2_ESM.docx]

**Table 1. Table of included studies (n = 49)**

| **Author**  **Year** | **Country** | **Design** | **Medical students; medical year; discipline** | **TBL or mTBL and elements missing** | **Outcomes and tools used** | **Key findings** |
| --- | --- | --- | --- | --- | --- | --- |
| Abouzeid et al.  2022 | Egypt | Prospective quasi-experimental study | 55; 5th year  Ophthalmology | Modified TBL. Peer review not described. Application exercise not described according to 4S. | Students’ perceptions assessed with anonymous questionnaire using Likert-type scale. Script concordance testing was performed individually and in teams. | Significant difference between students’ and experts’ test scores. When the test was completed in teams, the scores for 9 out of the 17 vignettes showed non-significant differences with the experts’ scores. |
| Alintas et al.  2014 | Turkey | Prospective cohort study | 169; 5th year  Ophthalmology | Modified TBL; missing 4S application exercises | Student satisfaction and engagement assessed with questionnaires using a 5-point Likert scale. | TBL sessions were considered better at fulfilling learning objectives, at understanding of the subject, ensured greater participation. |
| Anwar et al.  2015 | Saudi Arabia | Prospective cohort study | 156; 3rd year  Neuroscience/ neurology | Modified TBL; missing 4S application exercises and peer review of team members | Student learning and perceptions assessed by final exam grade and 24-item anonymous questionnaire using a Likert scale. | Students who attended TBl performed better. They had higher grades compared to previous block. High students’ satisfaction with TBL process. |
| Bergl et al.  2017 | USA | Prospective cohort study | 93; 4th year  Ambulatory medicine | Modified TBL; missing/not described peer review of team members | Student satisfaction. Evaluation questionnaires on the quality of the presenter, applicability of the presentation and cases, and preparatory materials; 5-point Likert scale and qualitative data from students' comments. | Student viewed TBL as high-quality presentation, applicable to their careers. They strongly agreed that case discussion facilitated deeper understanding on the subject and that the preparatory materials were adequate. |
| Borges et al.  2012 | USA | Prospective cohort study | 105; 3rd year  Internal medicine | Modified TBL; missing/not described peer review of team members | Team emotional intelligence pre- and post-clerkship assessed through workgroup emotional intelligence profile, short version (WEIP-S). | Team emotional intelligence increased significantly pre to post clerkship for three of the four areas: awareness of own emotions, recognizing emotions in others, and ability to manage other's emotions. There was no change for ability to control own emotions. |
| Carrasco et al.  2022 | USA | Retro-spective cohort study | 226; 1st/2nd year  Women health | Modified TBL. No peer-review described. | Knowledge assessed by iRAT scores. | Prior experience with TBL improves TBL iRAT scores, especially in struggling students. Prior TBL experience is associated with consistent iRAT performance in high performing students. |
| Daily et al.  2021 | USA | Prospective cohort study, descriptive | 336; 4th year  Sexual assault /psychiatry | Modified TBL; missing 4S application exercises and peer review of team members | Students' knowledge, attitudes, and confidence assessed by iRAT and questionnaires with a 5-point Likert scale. | Post TBL, students were confident in their ability to care for patients and friends who have survived sexual assault. |
| Fernandes et al.  2019 | USA | Cohort study | 123; 4th year  Clinical and communication skills | Hybrid method with simulated patients and TBL | Attitudes toward TBL evaluated using a 7-item questionnaire with a Likert scale and two qualitative items | Students had favorable attitudes towards TBL-simulated patients. |
| Field et al.  2019 | UK | Cohort study | 201; final year  Prescribing safety assessment | Modified TBL; no preparation phase and missing/not described peer review of team members | Self-rated confidence in prescribing and attitudes toward teamwork evaluated with a 16-item questionnaire with a 5-point Likert scale. | TBL improved attitudes towards “team experience”, “team impact on clinical reasoning and quality of learning. TBL increased prescribing confidence. |
| Huang et al.  2016 | China | Cohort study | 99; 8th year  Ophthalmology | Modified TBL; missing/not described peer review of team members | Knowledge acquisition and survey on self-perception of TBL evaluated with a 16-item questionnaire and rated using a 6-point Likert scale. Knowledge assessment with iRAT and gRAT, as well as final exam scores. | tRAT scores were higher than iRAT.57%students agreed that TBL helped with learning experience. The dissection lab, traditional lectures and textbooks rated higher than TBL. |
| Kek et al.  2019 | Canada | Cohort study | 360; 2nd year  Occupational medicine | Modified TBL; missing/not described peer review of team members | TBL experiences assessed through qualitative feedback. | Students gained good introduction to return-to-work issues; the process generated effective case-related discussions. |
| Langer et al.  2020 | USA | Cohort study | 136; 2nd year  Hematology | Modified TBL; missing/not described peer review of team members | Knowledge acquisition, students' evaluation, and facilitator feedback assessed through final exam scores, results on iRAT and gRAT as well as course evaluation. | The average score for iRAT was 63%. No difference between TBL and non TBL questions in final exam. |
| Lerchenfeldt et al.  2020 | USA | Cohort study | 342; 2nd year  Psychopathology | Modified TBL; missing/not described peer review of team members | Students' learning and quality of student learning assessed through iRAT, tRAT, and final exam statistics. Students' attitudes toward TBL assessed through a qualitative analysis of the course evaluation comments. | iRAT scores ranged from 80 to 88%. The averages on trat comparable over the three years. Course evaluations showed the TBL helped students think critically and integrate information. |
| Omer et al.  2021 | Saudi Arabia | Descriptive cross-sectional study | 34; 4th year  Surgery | Modified TBL; missing/not described preparatory phase | Students' satisfaction with accountability and preference for TBL assessed through a 33-item questionnaire (TBL–SAI) with a 5-point Likert scale. | Students expressed a minor level of accountability, poor preference and satisfaction and an overall low rating of TBL. |
| Smith et al.  2021 | USA | Cohort study | over 400; 3rd year  Family medicine | Modified TBL; missing/not described peer review of team members | Students' learning assessed through iRAT, tRAT, and team application exercise scores. Course evaluation assessed through an online survey. | Average iRAT scores were 80% and gRAT scores nearly 100%. Groups scored approximately 70% on the tAPP questions. Students rated the module on a 5-point Likert scale least 4. |
| Sward et al.  2019 | USA | Cohort study | 93; 3rd year  Obstetrics and gynecology | Modified TBL; missing/not described peer review of team members | Knowledge assessment and student satisfaction assessed through IRAT, gRAT, and student survey with a 5-point Likert scale and free comments. | Average iRAT scores were 88.9%, gRAT 98.8%. 98% students were satisfied with the teaching method. |
| Zgheib et al.  2011 | Lebanon | Cohort study | 127; 4th year  Internal medicine/clinical pharmacology | TBL | Knowledge assessment and students' satisfaction assessed through iRAT, gRAT, final exam scores, and a 15-item course evaluation with a 5-point Likert scale and comments. Faculty answered an 8-item questionnaire. | High students’ satisfaction. The students performed significantly better in prescription writing after last session TBL. Group performance on gRAT was significantly better than at iRAT. |
| **TBL with comparator** | | | | | | |
| Alimoglu et al.  2017 | Turkey | Prospective controlled follow-up study | 179 TBL/161 controls; 5th year  Neurology | Modified TBL–3S application exercises instead of 4S and missing peer review of team members | Knowledge retention, in-class learner engagement, and learner satisfaction assessed through student satisfaction scale, observation (STROBE), in-class engagement measure, end of clerkship exam scores, MCQ (10) one year later, iRAT, gRAT, and patient feedback forms for TBL only. | Higher mean satisfaction score and learner engagement for TBL. No difference between groups regarding end of clerkship exam scores. The knowledge retention in the TBL group was higher than the lecture group. The patients were highly satisfied. |
| Babenko et al.  2022 | Canada | Retrospective cohort study | 145/146/148; 3rd year  Family medicine | Modified TBL. No peer review described.  Application exercise not described according to 4S. | Knowledge assessed by iRAT, tRAT scores. The Generalized Estimating Equations analysis was performed | No significant IRAT-GRAT differences were observed between in-person and online delivery of TBL sessions. |
| Borges et al.  2015 | USA | Prospective cohort study | 484 TBL/265 controls; 3rd year  Psychiatry | Not described | Team emotional intelligence and team interactions assessed through WEIP-S and the Team Performance Scale (TPS). | Significant correlations existed between quality of team interactions and team emotional intelligence. Control and TBL groups experienced significant increases in WEIP-S subscales pre to post, with TBL group experiencing significantly higher gains in three of four subscales. Control group scored higher on TPS. |
| Boysen-Osborn et al.  2016 | USA | Cohort study, comparison with historic cohort | 95 TBL/259 controls; final year  Emergency medicine | Modified TBL; unclear if both iRAT and tRAT included in this TBL, missing /not described peer review of team members | Knowledge assessment through three written evaluations: MCQ test, cardiac rhythm test, and clinical management test. | For all tests combined median scores increased with TBL. More students failed 1 of 3 tests with lecture-based learning. |
| Brich et al.  2013 | Germany | Cohort study, pilot | 35 TBL/132 controls; 3rd year  Neurology | Modified TBL; missing/not described peer review of team members | Knowledge assessment, attitude toward TBL and neurology TBL assessed through final exam scores and written evaluation of TBL in general and of neurology TBL (6-point Likert scale). | TBL group had a higher overall total score and a significant better result in TBL topics. Favorable evaluation for TBL in general. |
| Brich et al.  2017 | Germany | Prospective crossover design with random distribution | 122 students (56 group A and 66 group B); 3rd and 4th year  Neurology | Modified TBL; missing/not described peer review of team members (sTBL) | Knowledge assessed by MCQ examination. Clinical reasoning assessed by key feature problem examination. Attitude toward TBL assessed through written evaluation based on the Trier Inventory for Teacher Effectiveness Evaluation and questionnaires with a 6-point Likert scale for further methodological evaluation. | No group differences in MCQ results. TBL better performance in the key feature in one of the TBL topics, no differences in other 3 topics. A clear majority of students preferred TBL as future teaching method. |
| Cevik et al.  2019 | United Arab Emirates | Cohort study with historic cohort as control | 66 TBL/79 controls; 6th year  Emergency medicine | Modified TBL; missing/not described peer review of team members | Knowledge retention and the experience of and personal perspectives on TBL assessed through final exam scores (exam different for the two cohorts) and 19-item survey with a 5-point Likert scale and two open-ended questions. | Student marks improved in the second year for both TBL and case discussion. The marks for topics taught via TBL showed better improvement. Marks also higher on the medical exit exam on these topics. Students’ response to TBL survey was positive. |
| Cremerius et al.  2021 | Germany | Prospective randomized trial | 19 TBL/36 peer-assisted learning/33 conventional teaching; 4th to 9th semester  Elective ultrasound skills | Modified TBL; no iRAT | Knowledge acquisition assessed through pretest and posttest and post-OSCE. Students' learning style assessed through Kolb's Learning Style Inventory (40 statements with a 4-point Likert scale). Course evaluation assessed through an 8-item survey with a 5-point Likert scale. | Significant gain in knowledge in all groups. Superior performance for TBL group in OSCE. Overall students were satisfied with all teaching methods. Learning style linked to differences in the practical outcome. |
| Faezi et al.  2018 | Iran | Quasi experimental design | 84; 3rd year (both interventions in the same group)  Rheumatology | Modified TBL; missing/not described peer review of team members | Students' attitudes and classroom engagement assessed using TBL–SAI, classroom engagement survey (CES), and short answer questions for assessment of knowledge. | Higher level of engagement with TBL. The effect of TBL on knowledge retention had become more pronounced over time. |
| Gong et al.  2022 | China | Randomized control study | 30;  Pediatrics | Modified TBL. Application exercise modified to a bedside task, not 4S. No peer-review described. | Knowledge assessed by computer-based case simulations. The examination of clinical performances was based on mini-Clinical Evaluation Exercise. Students ‘satisfaction assessed by survey with a five-point Likert scale. | Higher test scores for the TBL group. Clinical judgment and counseling skills of the intervention group was higher than controls. |
| Hashimi et al.  2014 | Pakistan | Comparative study | 72; 4th year  Community medicine | Modified TBL; missing/not described iRAT, tRAT, 4S application ex, and peer review of team members | Knowledge acquisition assessed through pre- and post-intervention knowledge test. Students' views on TBL assessed through an 11-item survey with a 5-point Likert scale. | Higher test scores after TBL compared to after lectures. Students thought that TBL was a better learning strategy compared to lectures. |
| Horne et al.  2017 | USA | Observational study | 30; 2nd and 4th year  Ophthalmology | Modified TBL; missing/not described peer review of team members | Self-reported satisfaction and learning experience, in comparison with team CBD, lecture. Outcomes assessed using 8 questions with a 5-point Likert scale and qualitative data from students' comments. | Students rated the team case-based sessions better with respect to the overall learning experience, enjoyment of learning, and increasing retention and ability to apply knowledge. |
| Huilaja et al.  2022 | Finland | Randomized control study | 50 pTBL/44 controls; 5th year  Pediatric and dermatological allergology | Modified TBL. Application exercises in peer led TBL and peer review not described. | Knowledge acquisition assessed by MCQ after the session and knowledge retention assessed by MCQ 5-6 months after. Students’ satisfaction by survey. | No difference in learning outcomes (immediate or long term) between the groups. pTBL was significantly preferred over faculty-led learning |
| Imran et al.  2022 | Saudi Arabia | Randomized cross-over design | 35/37; 3rd and 6th year  Endocrinology  /Emergency care | Modified TBL. Peer-review not described/absent | Learning assessed by MCQ test after the session. Clinical reasoning skills assessed with the same MCQ and students’ feedback by questionnaire. | TBL performed better in MCQs and in clinical reasoning questions. Most students satisfied with TBL. |
| Jost et al.  2017 | Germany | Static group comparison design, pilot | 11 TBL/15 controls; 4th and 5th year  Neurology | Modified TBL; missing/not described peer review of team members | Clinical decision-making skills using a key feature examination (voluntary for both groups) and factual and conceptual knowledge in MCQ tests (mandatory for both groups). | TBL group performed better in the key feature problem examination. No differences in the MCQ were found. |
| Kaminski et al.  2019 | USA | Implementation study, before and after | 199 TBL/208 controls; 3rd year  Surgery | Modified TBL; missing/not described peer review of team members | Knowledge acquisition assessed through NBME Surgery Subject Examination scores. Course evaluation assessed through the Association of American Medical Colleges (AAMC) Graduate Questionnaire. Students' perceptions of TBL assessed through qualitative feedback. | The mean score NBME decreased after the implementation. Students rated the surgery clerkship as good or excellent after TBL. Students found TBL effective, informative and as an opportunity for interactive learning. |
| Krase et al.  2018 | USA | Prospective cohort study | 247 students; pre-TBL, post-TBL  Obstetrics and gynecology | Modified TBL; missing 4S application exercises and peer review of team members | Knowledge acquisition assessed through National Board of Medical Examiners scores; knowledge retention assessed through voluntarily MCQ test. An online questionnaire issued by the University of Kansas School of Medicine was used to assess learner satisfaction. | Post TBL cohort performed better at NBME. No significant differences in student satisfaction. No differences in knowledge retention. |
| Langer et al.  2021 | USA | Cohort study | 70; 2nd year  Hematology | Modified TBL; missing 4S application exercises and peer review of team members | Knowledge acquisition and retention assessed through a test administered pre-course, post-course, and 14 months later. | Low pre-course knowledge. Higher scores for TBL post-course compared to control groups. No statically significant difference in long term knowledge retention. |
| Levine et al.  2004 | USA | Cohort study | 133 TBL/130 controls; 3rd year  Psychiatry | Modified TBL; missing/not described peer review of team members | Knowledge acquisition assessed through NBME psychiatry exam scores, students' engagement assessed through two items from the CES, Baylor College of Medicine. Students' perceptions of the value of learning in a team assessed through a 17-item "Value of Teams" survey with a 5-point Likert scale. | Higher scores at NBME in TBL groups. Students perceived team learning activities to be more engaging, effective, and enjoyable than conventional didactics. |
| Mayel et al.  2020 | Iran | Quasi experimental study | 33 TBL/32 controls  Emergency medicine | Modified TBL; missing/not described 4S application ex and peer review of team members | Knowledge acquisition assessed through pre- and post-intervention knowledge test. | No differences in pretest score between groups. TBL group scored higher in posttest. |
| Mody et al.  2013 | USA | Randomized control study | 69 TBL/61 controls; 3rd year  Obstetrics and gynecology | Modified TBL; missing/not described peer review of team members | Students' satisfaction assessed through a 3-item survey with a 5-point Likert scale. Knowledge acquisition assessed through pre- and post-intervention tests. | Both groups gained significant amount of knowledge with no differences between groups. TBL group reported that the learning style was a valuable experience, helped them learn the course material and improved problem-solving skills. |
| Ong et al.  2021 | Singapore | Crossover design | 179; 3rd and 5th year  Neurology | Modified TBL; missing/not described peer review of team members | Clinical reasoning assessed through script concordance test (SCT): 53 questions with 7 clinical scenarios. | TBL was superior to IL for teaching neurological localization, both methods were comparable for teaching neurological emergencies. |
| Salih et al.  2021 | Sudan | Cohort design | 176/202; 4th and respective 6th year 4 different topics: nephrotic syndrome, skin tumors, sickle cell anemia, brain tumors | Modified TBL. Feedback and discussion after tRAT not described and no peer review described. | Learning assessed by iRAT,tRAT and application exercises and for the control group a single best correct answer test. | Students’ performance using TBL was superior to lecture-based learning, irrespective of students’ gender. |
| Sannathimmappa et al.  2022 | Oman | Crossover interventional study | 139; 4th year  Immunology | Modified online synchronous TBL. No peer review described. | Knowledge assessed by pre- post-test. Students’ satisfaction assessed by online survey students’ feedback 3-point Likert scale. | Post test scores higher with TBL compared to self-study. High level of student satisfaction with TBL promoting active participation and engagement. |
| Saudek et al.  2015 | USA | Cohort design | 256 TBL/ 334 controls; 3rd year  Pediatrics | Modified TBL; missing/not described peer review of team members | Knowledge acquisition assessed through NBME pediatrics subject examination content area item analysis (CAIA) reports and examination of performance profiles (EPP). | Blood disorders domain scores from NBME pediatrics subject increased after the implementation of TBL. |
| Tan et al.  2011 | Singapore | Modified crossover study | 49; 3rd year  Neurology | Modified TBL; missing/not described peer review of team members | Knowledge acquisition and retention assessed through pretest; the second test was conducted after completion of the gRAT, and the third was conducted two days after the TBL. Student engagement assessed through self-reported student engagement STROBE with 5-point Likert scale. | Mean percentage change scores were greater in TBL vs passive learning in posttest 1 and 2. Academically weaker students showed greater increase in scores with TBL. High students ‘engagement with TBL. |
| Thomas et al.  2011 | USA | Unblinded crossover design | 112; 2nd, 3rd, and 4th year  Ambulatory medicine | TBL | Knowledge assessed through final exam scores, iRAT, and gRAT. Student evaluation of the sessions assessed through a global rating with a 5-point Likert scale. | Students in TBL group had higher scores on five out of 6 TBL topics. Students rated usefulness of the sessions equally. Positive comments on TBL. |
| Warrier et al.  2013 | USA | Cohort design | 179 (2009) + 160 (2010); 3rd year  Pediatrics | TBL | Students’ perception of the value of teamwork assessed through a nine-item "Value of Teams" survey. Students' engagement assessed through CES. Student evaluations. All items were scored on a 5-point Likert scale. Knowledge acquisition was assessed through NBME Pediatric Shelf scores, M4 Comprehensive Clinical Assessment (CCA), and a high-stakes OSCE. | Short term and long-term examination scores improved significantly. Lower satisfaction with TBL. Greater appreciation of teamwork after TBL, higher levels of engagement. |
| Wu et al.  2022 | China | Cohort study | 140 TBL/ 135 controls; 5th year  Ophthalmology | Modified TBL with an extended 1h lecture after tRAT. No peer-review described. | Knowledge assessed through iRAT,tRAT, scores from application exercises and final exam scores. Students’ satisfaction and evaluation of teaching goals assessed through questionnaires and interviews) | No significant difference in final exam scores. Higher results in gRAT compared to iRAT. Number of failed students higher in traditional lectures. Students satisfied with TBL. |
| Yang et al.  2014 | China | Comparison study | 41 TBL + LBL/43 LBL/43 TBL; 4th year  Neurology | TBL | Knowledge acquisition assessed through a theoretical and a practical test. Students' satisfaction assessed through a survey with a 4-point scale. | Group A (TBL+LBL) had highest scores. No significant difference between group B (LBL) and group C (TBL) in knowledge acquisition. Group C much lower practice scores than group B. Group A had 100 % satisfaction. |
| Zeng et al.  2017 | China | Randomized controlled trial | 55 TBL/56 controls; 3rd year  Clinical medicine | Modified TBL; not clearly described 4S application exercises | Knowledge acquisition and retention assessed through test 1 after class and test 2 after one week. Students' attitudes toward TBL assessed through a 10-item survey with a 5-point Likert scale. Teachers' teaching efforts and attitudes toward TBL were assessed through interviews. | No differences in scores on test 1 between groups. For test 2 the scores of TBL were significantly higher. The survey showed that students evaluated TBL teaching model positively. |
| Zeng et al.  2021 | China | Cohort study | 52; 5th year  Dermatology | Modified: Small private online course (SPOC) and TBL | Module effectiveness and students' satisfaction assessed through surveys. Knowledge acquisition assessed through final exam scores. | Higher scores on the case analysis questions in the final theoretical examination for the experimental group. High students’ satisfaction. |
